# Deep Sclerectomy with Porous Collagen in Open-angle Glaucoma, Short-term Study

**DOI:** 10.5005/jp-journals-10008-1249

**Published:** 2018-08-01

**Authors:** Ahmed Elbably, Tageldin M Othman, Ahmed Mousa, Mohammed Elridy, Wesam Badawy, Mohammed Elbably

**Affiliations:** 1 Anterior Segment and Glaucoma Specialist, Magrabi Eye Hospital, Riyadh, Kingdom of Saudi Arabia; 2 Lecturer of Ophthalmology, Glaucoma and Anterior Segment Consultant, Aswan University, Aswan, Egypt and King Saud Medical City, Riyadh, Kingdom of Saudi Arabia; 3 Founder and Chairman, Nourseen Charity Foundation for Community Ophthalmology Cairo, Egypt; 4 Anterior Segment and Glaucoma Fellow, Magrabi Eye Hospital, Riyadh, Kingdom of Saudi Arabia; 5 Anterior Segment Specialist, Jeddah Eye Hospital, Jeddah, Kingdom of Saudi Arabia; 6 Anterior Segment Specialist, Al-Adan Hospital, Kuwait, Kuwait

**Keywords:** DS, Glaucoma, Ologen.

## Abstract

**Aim:**

To assess the role of porous collagen in deep sclerectomy (DS), with and without trabeculo-Descemet membrane (TDM) rupture.

**Patients and methods:**

Forty-six eyes with different types of open-angle glaucoma and medically uncontrolled intraocular pressure (IOP) were selected. DS was performed in all cases. Ologen was implanted as a single large piece in the scleral lake and subconjunctival space in all cases with and without TDM rupture.

**Results:**

A total sample of 46 open-angle glaucoma patients were included in the study. The mean ± standard deviation (SD) IOP was 25.6 mm Hg ± 10.6 (range 12-58 mm Hg) pre-operatively. On follow-up, the mean ± SD IOP was 6.1 mm Hg ± 3.7 (range 2-20 mm Hg) 1 day postoperatively and 9.3 mm Hg ± 4.0 (range 4-23 mm Hg) after 1 month of follow-up, at 12 months the IOP was at 12.1 mm Hg ± 3 (range 8-18 mm Hg). The overall mean ± SD IOP reduction percentage was 48.3% ± 21.3 (range 0.0-86.2). Comparing mean IOP reductions at last follow-up between TDM rupture cases and non-TDM cases (Mann-Whitney test), the mean ± SD IOP reduction in TDM rupture patients was 12.1 ± 8.0 mm Hg (range 2-27) meanwhile, in non-TDM rupture patients it was 14.3 ± 11.4 mm Hg (range 0-50). However, the difference in IOP reduction between the two groups was not significant. (p = 0.689)

**Conclusion:**

Porous collagen can enhance the results of DS; also, it helps to proceed with DS in cases of TDM rupture without converting to trabeculectomy.

**How to cite this article:** Elbably A, Othman TM, Mousa A, Elridy M, Badawy W, Elbably M. Deep Sclerectomy with Porous Collagen in Open-angle Glaucoma, Short-term Study. J Curr Glaucoma Pract 2018;12(2):85-89.

## INTRODUCTION

DS surgery was designed to increase the success rate and lower the risk of complications of trabeculectomy, thus, giving both patient and surgeon a safer and more convenient option.^1,2^

Four possible mechanisms of decrease IOP can be achieved in DS: (1) subconjunctival bleb, (2) scleral lake, (3) suprachoroidal space and (4) episcleral venous outflow. For the sake of improving the success and reducing complications, different modalitieshave been used includingthe use of antimetabolites. In addition, glaucoma implants have been used to increase the success rates of DS.^1-9^

Space maintaining implants in the scleral lake were first tried by Kozlov.^2^ Since then, non-absorbable, absorbable, and autologous implants were the main kinds being used till now. The first implant was a purified porcine collagen (Aquaflow®, Staar Surgical AG) which takes six to nine months to be absorbed. Later on, reticulated hyaluronic acid implants (SK-GEL®, Corneal) were used. Recently, a semi-solid-cross-linked sodium hyaluronate implant (Healaflow®) has been injected inside the scleral lake and subconjunctivally.^2,10,11^

Despite the various surgical techniques and implants for DS, the perfect solution has not yet been met, and no study shows the superiority of any of the commercially available implants over the others. Future studies are needed to compare different types of DS implants regarding their safety and efficacy.

In June 2002, a study was published in the Journal of Glaucoma aimed to assess the safety and efficacy of deep sclerectomy with collagen implant in one eye and trabeculectomy in the other eye of the same patient. This study concluded that deep sclerectomy with collagen implant is comparable with trabeculectomy in decreasing the intraocular pressure but with a less rate of early postoperative complications. According to this study, The IOP was decreased by 39.7% in the deep sclerectomy with collagen implant group at 24 months, and by 55.9% in the trabeculectomy group.^12^ Many studies show that DS controls the IOP in the early postoperative period. However, without DS implants, the IOP-control fails over the long term.^12^

Ologen (Aeon Astron Europe B.V.), a porous collagen material that is thought to modulate the wound healing.

Theoretically, it functions on a physical basis rather than a chemical one. The possible mechanism of action is not by suppressing fibroblast growth but by random physiological growth within the matrix, without scar formation, forming a bleb without hypotony. This spongy collagen is thought to biodegrade within 3-6 months eventually. The main Ologen studies showing efficacy are currently underway. Additionally, in one study, the complete success rate using the Ologen implant in trabeculectomy was found to be lower than that of trabeculectomy with Mitomycin C.^13^

Furthermore, follow-up results confirmed that the success rates of an Ologen implant in trabeculectomies were quite similar to those of MMC with trabeculectomies.^14^ Ologen can be used in DS, bleb revision, and drainage implantation surgeries.

Therefore, there is a need to conduct a study to assess the role of Ologen implant in DS.

## PATIENTS AND METHODS

### Case selection

Forty-six eyes with different types of open-angle glaucoma and medically uncontrolled IOP were selected consecutively to be recruited in the current study, and were then randomly assigned to our interventions. The study patients were encountered after the approval of the ethics committee of Magrabi Eye Hospital in Riyadh, Kingdom of Saudi Arabia. Informed consent was obtained from all patients. Encountered patients were all with uncontrolled IOP with maximal tolerable medical therapy. Uncontrolled glaucoma was defined as uncontrolled IOP (≥22 mm Hg) measured with a Goldmann applanation tonometer under maximal tolerable medical treatment with visual field defect progression. Exclusion criteria were known allergy to collagen, history of surgery in less than 6 months before enrolment in this study and patient refusal.

### Surgical procedures

Deep sclerectomy with Ologen which was implanted as a single large piece in the scleral lake and subconjunctival space.

### Deep sclerectomy

**Fig. 1:**
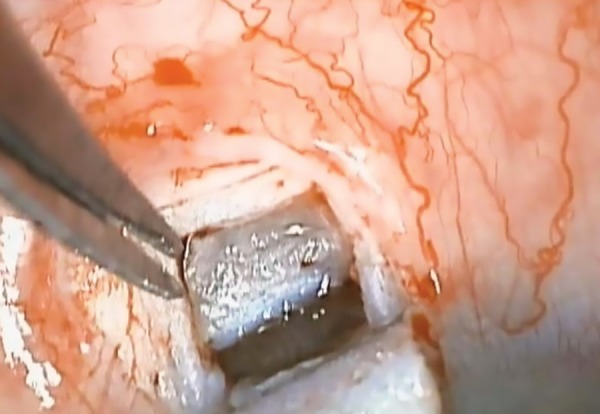
Deep sclerectomy with wide trabeculodescemet window

**Fig. 2:**
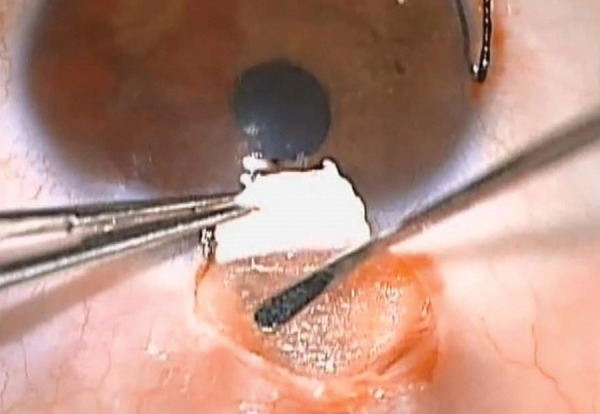
Ologen in both scleral lake and subconjunctival space

A one-third scleral-thickness scleral flap measuring 4x4 mm was fashioned 1 mm into clear cornea. Another deep flab 3x3 mm of deep sclera was created, MMC 0.2 mg/ ml was applied for two minutes. Anterior dissection was performed reaching the Schlemm’s canal, which was unroofed. Then, the creation of the TDM membrane was done, followed by the excision of the deep scleral flap. Different sizes and shapes (rectangular 3 mm × 5 mm, mushroom and circular of 12 mm diameter) of a large single piece of the collagen implant (Ologen, spongy collagen that contains a connected porous structure of 10-300 μm diameter made of cross-linked lyophilized porcine type I atelocollagen (≥ 90%) and glycosamino-glycans (GAG) (≤ 10%) were then implanted in the scleral bed and the subconjunctival space. The superficial scleral flap was repositioned and left without suturing (Figs 1 and 2).

Surgery was considered a complete success when IOP was < 22 mm Hg without glaucoma medication and a qualified success when IOP was < 22 mm Hg with glaucoma medication. It was considered a failure when IOP was ≥ 22 mm Hg with glaucoma medication, or when an eye required further glaucoma drainage surgery.

Goniopuncture with the Nd: YAG laser was performed when the filtration through the trabeculo-Des-cemet’s membrane TDM was suspected to be insufficient, because of increased IOP. Laser goniopuncture would be successful when final IOP was ≤ 17 mm Hg, or when the reduction in IOP was ≥ 6 mm Hg.

Data recorded preoperatively before surgery, IOP was measured using a Goldmann applanation tonometer. Patients also underwent angle examination using gonioscopy, visual field testing and fundus examination. Following surgery, all these examinations, apart from the visual field, were performed on the first day and then repeated after one week as well as in 1, 3, 6, 9, and 12 months. Visual field was repeated every 6 months.

Complications were considered as follows- Hypotony was considered when a postoperative IOP < 5 mm Hg for more than 2 weeks. The anterior chamber depth was clinically compared with the fellow eye. Anterior chamber was shallow when a peripheral iridocorneal touch occurred and flat with a lenticulo corneal touch. Anterior chamber flare would indicate anterior chamber inflammation. Choroidal detachment would be present when seen in the peripheral retina by an indirect ophthalmoscope.

### Statistical Methods

A specific data collection sheet was used to collect data, pre- and post-operatively, data were then cleaned, managed, and coded using Microsoft Excel 2013®; Microsoft Corporation, Redmond, Washington, USA. SPSS® version 22 (IBM Inc., Chicago, Illinois, USA) was used to perform the analysis.

Descriptive analysis was done, where categorical variables were presented in the form of frequencies and percentages while continuous variables in the form of the mean (±SD). Inferential analysis was conducted to test the significance of potential change between pre- and post-OCT measurements. Testing the difference in IOP reduction between groups was done by Mann-Whitney test. Ap below 0.05 was considered as an indicator of statistical significance.

## RESULTS

**Fig. 3:**
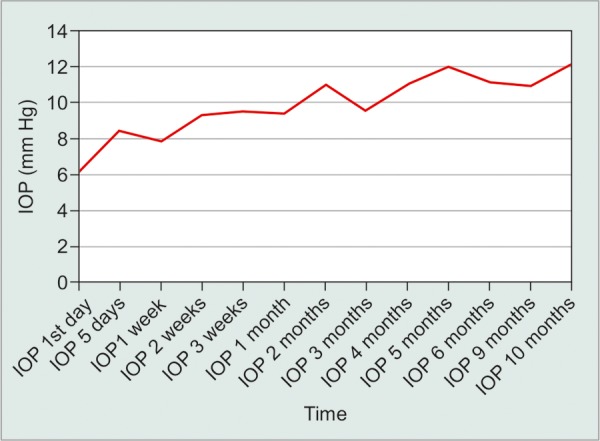
IOP periodic readings

A total sample of 46 open-angle glaucoma patients was included in the study, where 38 (82.6%) were males and 8 (7.8%) were females. The mean ± standard deviation (SD) age in years was 44.7 ± 19.9, range 4 months to 71 years. The mean ± SD IOP was 25.6 mm Hg ± 10.6 (range 12-58 mm Hg) preoperatively (Table 1). On follow-up, the mean ± SD IOP was 6.1 mm Hg ± 3.7 (range 2-20 mm Hg) Day 1 postoperatively and 9.3 mm Hg ± 4.0 (range 4-23 mm Hg) after 1 month of follow-up. At 5 months, mean ± SD IOP was 12 mm Hg ± 3.9 (range 7-18 mm Hg), this remained stable at 12 months with IOP at 12.1 mm Hg ± 3 (range 8-18 mm Hg). The overall mean ± SD IOP reduction percentage was 48.3% ± 21.3 (range 0.0-86.2) (Fig. 3).

At 12 months, the complete success rate (IOP < 21 mm Hg without medication) was 100%. Additionally, the mean ± SD number of medications per patient, which was 1.6 ± 1.4 (range 0-4) preoperatively, was zero postoperatively.

**Table 1:** **Table 1:** Demographics and patients characteristics

*Characteristic*		*N (%)*	
*Eye*			
OD		22 (47.8)	
OS		24 (52.2)	
Age in years, Mean ± SD (Range)		44.7 ± 19.9 (4	
		months-71 years)	
*Gender*			
Male		38 (82.6)	
Female		8 (17.4)	
Pre-op IOP (mm Hg), Mean ± SD (range)		25.0 ± 10.6 (12-58)	
Pre-op Medications, Mean ± SD (range)		1.6 ± 1.4 (0-4)	
*Diagnosis*			
OAG		35 (76.1)	
Congenital glaucoma		5 (10.9)	
Pigmentary glaucoma		2 (4.3)	
Aphakia		1 (2.2)	
NTG		1 (2.2)	
Steroid induced		1 (2.2)	
Uveitic glaucoma		1 (2.2)	

Of the 46 patients, over a period of 12 months, no patient underwent reoperation or trabeculectomy.

**Fig. 4:**
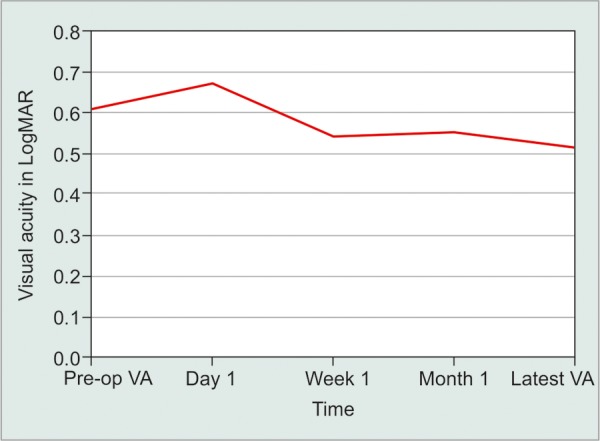
Visual acuity periodic readings

The mean ± SD visual acuity in logMAR was 0.61 ± 0.65 preoperatively and 0.67 ± 0.611 days postoperatively. Visual acuity improved after preoperative levels 1 week after surgery and slightly improved over the next 12 months with a mean ± SD of 0.52 ± 0.63 at 12 months (Fig. 4).

The major intraoperative complications were perforation of the TDM during the DS dissection 11 cases 23.9%, two cases of iris prolapse 4.3% and one case of mild leakage 2.2%.

Comparing mean IOP reductions at last follow-up between TDM rupture cases and non-TDM cases (Mann-Whitney test), the mean ± SD IOP reduction in TDM rupture patients was 12.1 ± 8.0 mm Hg (range 2-27) meanwhile, in non-TDM rupture patients it was 14.3 ± 11.4 mm Hg (range 0-50). However, the difference in IOP reduction between the two groups was not significant (p = 0.689).

Regarding the postoperative complications: no shallow or flat anterior chamber, nobleb-related endophthalmitis, or surgery-induced cataract. However, Nd: YAG laser goniopuncture was performed in 6 patients (13%) and IOP reduction after YAG goniopuncture mean ± SD percentage was 48.2% ± 27.0 (range 10.0-77.8)

## DISCUSSION

Although standard trabeculectomy is an effective surgery to lower IOP, most of the surgeons prefer to delay surgery because of its potential complications.

Shaarawy et al. confirmed that the use of a collagen implant in DS increases the success rate and decrease the need for postoperative medications.^8^ Sanchez et al.^15^ also stated that collagen implant would provide a better surgical outcome.

Various techniques of non-perforating glaucoma surgery have been designed to avoid the postoperative complications of trabeculectomy. Zimmerman et al.^16,17^ stated that non-penetrating glaucoma suregery was effective in both phakic and aphakic patients.

Non-penetrating glaucoma surgery prevents the occurrence of early postoperative hypotony that commonly occurs after trabeculectomy by controlling the filtration of aqueous humor from the anterior chamber to the subconjunctival space, without perforation of the eye. At this stage, DS was safe but not that effective. To enhance the results, Stegmann et al.^18^ were the first to design viscocanalostomy, in which they filled the sch-lemm’s canal and the scleral space with a viscoelastic material, and they reported a complete success rate of 82.7% and qualified success in 89.0% of the patients at 35 months follow-up.

Also, in a trial aimed to increase the success rate of non-penetrating glaucoma surgery, Kozlov et al.^2^ described the use of a collagen implant. On the same line, Dahan used an unabsorbable implant (unpublished data); meanwhile, Stegmann et al. assigned high viscosity hyaluronic acid.^18^

Chiou et al.^3,4^ reported that ultrasonic biomicroscopy findings revealed that the scleral lake was kept open by the presence of the collagen implant.

Experimentally, Vaudaux et al.^19^ studied the aqueous dynamics through the TDM membrane. They discovered that the outflow resistance was low but sufficient to control the IOP and to prevent early hypotony.

In the literature, DS with collagen seems to be as effective as trabeculectomy and by far safer, but it is not that popular. A survey on 252 American Glaucoma Society members stated that trabeculectomy with MMC is the most popular primary penetrating surgery when performed alone or in combination with phacoemul-sification cataract extraction. Meanwhile, DS was not even mentioned in the survey which indicates that it is not one of the favorite glaucoma surgeries for glaucoma surgeons.^20^ The steep learning curve is the main reason for not being a popular surgery.

The steep learning curve of DS is one of the major difficulties facing many of glaucoma surgeons. Rupture of the TDM is the most common complication facing juniors as well as some experts; it forces them to convert DS into trabeculectomy. This explains why DS is not that popular and not one of the favorite glaucoma surgeries.

In that context, we aimed in our study to assign a glaucoma surgery technique, which is as effective as trabeculectomy, as safe as DS and without a steep learning curve.

Porous collagen guides fibrosis to occur within the mini pores of the collagen, then when the collagen biodegrades after six months, new mini porous fibrous meshwork will be already created, which will act as a flow controller, decreasing the risk of hypotony.

As per our short-term study results, our technique is as effective as DSCI DS with collagen implant, as the overall IOP reduction percentage in both was almost similar. Unlike DS with collagen implant, ologen was implanted in subconjunctival space and in scleral lake as well.

To evaluate the safety of our technique, the postoperative complications and the final outcomes in cases without TDM rupture were comparable to those in patients who had DSCI. Whereas, the major advantage of our technique over the DSCI is safety in patients who had TDM rupture, as no need to convert DSCI into trabeculectomy.

In trabeculectomy, several techniques and tricks are needed to control the outflow. Then, in DSCI with TDM rupture, it is mandatory to master those techniques and tricks, as DSCI will be converted into trabeculectomy. But in our study, it is that simple to control the outflow by just placing a large piece of ologen over the perforation site. This solves the problem of a DS steep learning curve (TDM rupture).

Depending on the criteria of success rate, the short-term success rate of our study at 12 months was 100% complete success as all the cases had IOP less than 21 mm Hg without medications. In our study, except for the first postoperative week, visual acuity was not affected. Furthermore, visual acuity remained stable as no surgery-related cataracts developed.

Perforation of the thin TDM during the DS dissection 23.9% was considered the main intraoperative complication. This was common in the learning phase of DS. The complication rate sharply decreases as surgical experience increases. Iridectomy was performed once the TDM perforation was large. Small and large perforations were both managed by applying a large piece of ologen over the perforation site, which made this technique easier.

Comparing mean IOP reductions at last follow-ups between TDM rupture cases and non-TDM rupture cases were not significant. Therefore, in TDM rupture cases, ologen acts as a new permeable membrane repairing the perforation and controlling the outflow.

Thirteen percent of patients required an Nd: YAG goniopuncture. Tha significant IOP reduction following Nd:YAGgoniopuncture made the success rate satisfactory. Thus, DS became a perforating filtration procedure.

As the incidence of late bleb-related endophthal-mitis may increase after goniopuncture, no cases were detected.

Some early postoperative complications- (a) Hypotony without maculopathy which lasted for a few days. (b) Mild wound leakage, which was controlled by bandage contact lens for a few days. (c) Small iris prolapse following TDM rupture or following YAG goniopuncture, it was managed by applying YAG laser over the prolapsed iris. Miotics did not work properly.

## CONCLUSION

In conclusion, porous collagen in DS acts as a space maintainer and a flow controller as well. Therefore, its major advantage over other collagen implants is that it can manage the most common intraoperative complication of DS (TDM) easily, safely and effectively. This would help deep sclerectomy to become more popular and adopted by many glaucoma surgeons.
